# Validation of a multicellular tumor microenvironment system for modeling patient tumor biology and drug response

**DOI:** 10.1038/s41598-021-84612-z

**Published:** 2021-03-10

**Authors:** Devin G. Roller, Stephen A. Hoang, Kristopher D. Rawls, Katherine A. Owen, Michael B. Simmers, Robert A. Figler, Julia D. Wulfkuhle, Emanuel F. Petricoin, Brian R. Wamhoff, Daniel Gioeli

**Affiliations:** 1 Departments of Microbiology, Immunology, and Cancer Biology, University of Virginia, MR6 Rm B526 345 Crispell Dr, PO Box 801318, Charlottesville, VA 22908 USA; 2 Cancer Center Member, University of Virginia, Charlottesville, VA USA; 3 HemoShear Therapeutics, Inc., 501 Locust Ave #301, Charlottesville, VA 22902 USA; 4 Center for Applied Proteomics and Molecular Medicine, George Mason University, Manassas, VA USA

**Keywords:** Cancer, Cancer microenvironment, Cancer models, Lung cancer

## Abstract

Lung cancer rates are rising globally and non-small cell lung cancer (NSCLC) has a five year survival rate of only 24%. Unfortunately, the development of drugs to treat cancer is severely hampered by the inefficiency of translating pre-clinical studies into clinical benefit. Thus, we sought to apply a tumor microenvironment system (TMES) to NSCLC. Using microvascular endothelial cells, lung cancer derived fibroblasts, and NSCLC tumor cells in the presence of in vivo tumor-derived hemodynamic flow and transport, we demonstrate that the TMES generates an in-vivo like biological state and predicts drug response to EGFR inhibitors. Transcriptomic and proteomic profiling indicate that the TMES recapitulates the in vivo and patient molecular biological state providing a mechanistic rationale for the predictive nature of the TMES. This work further validates the TMES for modeling patient tumor biology and drug response indicating utility of the TMES as a predictive tool for drug discovery and development and potential for use as a system for patient avatars.

## Introduction

Cancer drug development is limited by the lack of efficiency in translating pre-clinical studies into clinical benefit^1,2^. Greater than 95% of oncology drugs that progress through pre-clinical studies fail in clinical trials^3^. Therefore, it is essential to improve the accuracy of evaluating pre-clinical drug efficacy. To develop a more predictive translational pipeline, we have focused on developing more physiologically-relevant human models. Traditional in vitro systems do not accurately predict the efficacy of drug therapies^1,4^. This is unsurprising when considering the plethora of differences between the environment of cancer cells grown in static 2D cultures and the complex tumor microenvironment (TME) present in patient tumors. The TME contains a multitude of cell types, including vascular endothelial cells, fibroblasts, immune cells, and a complex 3D extracellular matrix (ECM). All of these cell types and architecture contribute to the proliferation and survival of cancer cells and serve as potential targets for therapeutic intervention^5^. New promising in vitro models are being developed to address the deficiencies in the current systems. These include spheroids, organoids, organotypic tumor models, and ex vivo systems^6^. However, these systems still do not fully capture the complement of cancer biology, which includes the contribution of vascular endothelial cells under physiologic shear.

In vitro 3D systems derived from patient tumors, human-induced pluripotent stem cells, and murine cells have been used for drug screening and to identify molecular mechanisms of disease progression^7,8^. 3D spheroids are the most commonly used 3D culture systems that have been used in the preclinical therapeutic screening setting^9^. These systems have clear advantages over standard 2D static tissue culture, but like all model systems, there are limitations. For example, the generation of spheroids often uses exogenous extracellular matrix such as Matrigel. Matrigel has been plagued with batch-to-batch variability and contamination with lactate dehydrogenase-elevating virus^10^. The media formulation for 3D organoids from primary tissue is often complex requiring the addition of multiple growth factors and the ROCK inhibitor. Furthermore, these systems lack vascular systems that recapitulate oxygenation, nutrient-delivery, and waste removal^11^. Certain 3D systems will use microvessel fabrication to generate a vascular system, through a predefined ECM scaffold or by enabling microvessel self-assembly^12–16^. However, these systems create disordered, non-physiologic flow due to restricted vessel size and random vessel networks. The tumor microenvironment assessed by histologic analysis is preserved in e*x vivo* explant systems^8,17,18^. However, these explants survive only for a few days and the most sophisticated ex vivo systems require a mixture of autologous and exogenous serum, and growth factor supplementation. While these ex vivo systems improve retention of the cancer tumor microenvironment, they still lack integration of vascular shear and biological transport. In vivo xenografts from long-cultured tumor cell lines have a poor correlation with human clinical outcomes, and it is still being determined how robust the predictive value is of patient-derived xenografts (PDXs)^4,19,20^. PDXs preserve the histopathology, tumor heterogeneity, gene expression and genetic mutant drivers^21,22^, however, PDXs take considerable time and cost to generate, have low tumor take rates, and undergo selection for the most aggressive tumor subtypes^23^. Furthermore, PDXs can undergo significant selection of preexisting sub-clones driven by the mouse microenvironment^24^.

We recently developed a multi-cellular 3D in vitro system for pancreatic cancer^25^. This tumor microenvironment system (TMES) combines human primary microvascular endothelial cells (ECs) exposed to tumor hemodynamics, human primary pancreatic stellate cells, and human PDX-derived pancreatic tumor cells. In that study, we demonstrated that significant tumor cell transcriptomic changes occur in the TMES which correlate with the in vivo xenograft and patient transcriptome, and that treatment with therapeutically relevant doses of chemotherapeutics yields responses paralleling the patients’ clinical responses. Here we explicitly tested if the TMES could be applied to another solid cancer to generate a patient-like in vivo state and response to therapeutics at clinically relevant doses. The rate of lung cancer is rising globally and it is the most frequent cause of cancer death in men and women^26^. Non-small cell lung cancer (NSCLC) represents 85% of lung cancers, with a five-year survival rate of only 24% (American Cancer Society 2020). Using microvascular endothelial cells, lung cancer derived fibroblasts, and NSCLC tumor cells in the presence of patient tumor-derived hemodynamic flow and transport we demonstrate that the TMES predicts drug response to EGFR inhibitors, a standard of care for EGFR mutant NSCLC. Transcriptomic and proteomic profiling indicate that the TMES recapitulates the in vivo xenograft and patient molecular biological state providing a mechanistic rationale for the predictive nature of the TMES. This work further validates the TMES for modeling patient tumor biology and drug response indicating utility of the TMES as a predictive tool for drug discovery and development, as well as the study of the underlying biology of cancer.

## Materials and methods

### Cell culture

Primary human microvascular endothelial cells (ECs) were purchased from PromoCell (Heidelberg, Germany) and maintained up to 8 passages in Endothelial Cell Growth Media MV2 (PromoCell, C-22121). Primary human lung fibroblast cells (Hs888lu) were purchased from ATCC (Manassas, VA) and maintained up to 8 passages in DMEM high glucose (Gibco, Gaithersburg, MD) supplemented with 10% fetal bovine serum (Gemini, Sacramento, CA) and 1% L-Glutamine. NSCLC cell lines, A549, H1975, and H1650 were purchased from ATCC. Firefly luciferase positive NSCLC cells were generated by transduction with lentivirus containing pLenti CMV Puro LUC (addgene #17477) at 10 MOI. Virus was generated as previously described^27^. A549 were maintained in DMEM high glucose and supplemented with 10% FBS and puromycin selection. H1975 and H1650 were maintained in RPMI (Gibco), plus 10% FBS, 1% sodium bicarbonate (Gibco), and puromycin selection.

### Patient derived xenografts and cell line xenografts

TM00199 and TM00219 PDXs were purchased from The Jackson Laboratory (Bar Harbor, ME). The PDXs and NSCLC cell lines xenografts were grown subcutaneously in NSC mice (The Jackson Laboratory) in compliance with the relevant laws and institutional guidelines with approval of the University of Virginia Animal Care and Use Committee. The day of experimental plating PDX tumors were dissociated to a single cell suspension using the Tumor Dissociation Kit (Miltenyi Biotec, 130-095-929), followed by the Mouse Cell Depletion Kit (Miltenyi Biotec, 130-104-694) and Debris Removal Solution (Miltenyi Biotec, 130-109-398) to enrich for PDX cells. All kits were used following the manufacturer’s instructions.

### Tumor microenvironment system

The transwell co-culture plating and hemodynamic flow device setup was previously described in detail^25^. A 0.4 μm pore polycarbonate 75 mm transwell membrane (Corning Inc, Corning, NY) was coated with 500 μl of 0.1% gelatin on the top and 2 mg/ml collagen on the bottom surface of the membrane prior to cell plating. Hs888lu cells were co-plated with NSCLC tumor cell line on the underside of the transwell at 11,363 cells per cm^2^ and 34,090 cells per cm^2^, respectively and allowed to adhere for 1 h. For studies when RNA, DNA, and protein collection was the primary readout, a negative plating mask was used during plating that enables plating of the cancer associated fibroblast on 64% of the membrane but occludes the fibroblasts from 9 precise 15 mm diameter areas representing 36% of the membrane that can then be cut at the end of an experiment^25^. ECs were plated on the upper side of the membrane at a density of 50,000 cells per cm^2^. All cells were plated in M199 (Gibco) supplemented with 10% FBS, 1% L-Glutamine, 1% Penicillin–Streptomycin, and 2.5% HEPES. Media used for plating ECs was supplemented with 0.1% Endothelial Cell Growth Supplement (ECGS). Cells were incubated overnight in a humidified chamber at 37 °C with 5% CO_2_. Following overnight incubation, plating media was aspirated from the transwell, cells were washed twice with phosphate-buffered saline, and media was replaced with flow media containing reduced serum and dextran (M199 supplemented with 5% Dextran (Pharmacosmos, Denmark), 2% FBS, 1% L-Glutamine, 1% Pencillin-Streptomycin, and 2.5% HEPES). Tumor hemodynamic and flow procedure was previously described^25^. Inflow and outflow tubing accessing the upper and lower chambers of the transwell allowed for continual perfusion of media into both the EC and tumor/fibroblast sides; the inflow media flow rate of 52.0 μl/min and outflow flow rate of 62 μL/min provide continuously exchanging media and creates an equilibrium of 4 ml in the upper chamber and 9 ml in the lower chamber. Cells were grown under hemodynamics for 7 days. For drug studies, all drugs were added into the flow media of the top chamber on days 4–7. The Luciferase Assay System (Promega, E1500), which served as a surrogate readout of tumor cell growth, was used according to the manufacturer’s instructions. Luciferase positive tumor cells were counted, pelleted, and snap frozen during plating of each device experiment to serve as a standard curve. For isolation of RNA, DNA, and protein, cells were scraped from the transwell in PBS, centrifuged to pellet, and flash frozen. TRIzol (ThermoFisher) was added to cell pellets and RNA, DNA, and protein were extracted according to the manufacturer’s instructions.

### Transcriptomic analysis

For each NSCLC cell line (A549, H1975, H1650) grown on device, in 2D static conditions, and as xenografts (n = 6 for each growth condition), and PDX (TM00199 and TM00219) grown on device, and as xenografts (n = 6 for each growth condition) all raw RNA-seq datasets were pseudoaligned to the human (GRCh38) and mouse transcriptomes (GRCm38) simultaneously using salmon v0.9.1^28^. Alignments to mouse transcripts were discarded in downstream analyses. Gene-level quantifications were calculated using the Tximport tool with the “LengthScaledTPM” option, and Ensembl gene and transcript annotations^29,30^. Library sizes were adjusted using the TMM normalization method^31^. Extra-experimental heterogeneity was identified in the data using the surrogate variable analysis (SVA) algorithm^32^. Differential expression analysis was performed independently on each cell line using the edgeR Bioconductor package^33^. The surrogate variables identified by SVA were included as nuisance variables in the design matrices used in the differential expression analysis.

TCGA Pan-Cancer Atlas transcriptomic data was downloaded via the cBioPortal^34,35^. Primary lung adenocarcinoma data in the database were retrieved and matched to our cell lines on the basis of their mutational status. The TCGA data was quantile normalized with the cell line data for downstream analyses. Contrasts between the TCGA samples and the cell lines were evaluated with the Mann–Whitney U test.

For each contrast in the differential expression analysis, a set of probabilities was calculated, corresponding to the Bayesian posterior probability that the null hypothesis is false (i.e., the posterior probability that the gene is differentially expressed)^36^. When comparing two different contrasts we calculated a value referred to as the response similarity index (RSI) for each gene. It is defined as the joint posterior probability of differential expression, signed by the sign of the product of the log fold changes in the two contrasts^25,37^. Thus, values near + 1 indicate joint differential expression in the same direction (concordance), values near − 1 indicate joint differential expression in opposite direction (discordance), and values near 0 indicate lack of joint differential expression.

The limma Bioconductor package was used to process and analyze publicly available microarray data^36,38^. Specifically, normalization and probe-level summaries were calculated using the Robust Multiarray Average (RMA) technique implemented in limma. Differentially expressed genes were identified by fitting linear models to each probe set and calculating p-values from limma’s moderated t-statistic. Subsequent processing and analyses followed the same procedure as the RNA-seq data.

Gene set-level quantifications used in pathway-level visualizations were generated using the Gene Set Variability Analysis (GSVA) method^39^. Gene set enrichment analyses based on RSI values were performed using the rank-based method implemented in the ‘geneSetTest’ function in the *limma* Bioconductor package^40^.

### Reverse phase array analysis

Reverse phase protein array (RPPA) was performed as previously described^25,41–44^. For each NSCLC cell line (A549, H1975, H1650) grown on device, in 2-D static conditions, and as xenografts (n = 6 for each growth condition), and PDX (TM00199 and TM00219) grown on device, and as xenografts (n = 6 for each growth condition), protein was extracted using TRIzol according to the manufacturer’s instructions. Protein was then diluted in 2 × Tris–glycine SDS sample buffer (Life Technology Corporation, Carlsbad CA) before printing on nitrocellulose slides (Grace Bio-Labs, Bend OR) and spotted in triplet with Auschon 2470 contact pin arrayer (AushonBioSystem Inc., Billerica MA), in 4-point two-fold curves.

## Results

**Figure 1:**
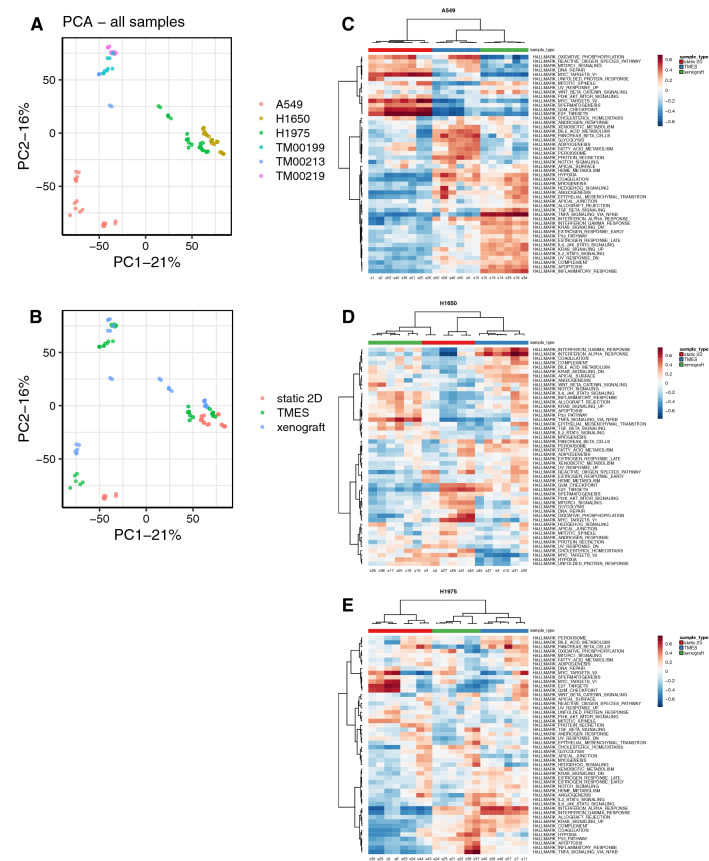
Transcriptomic Analysis of NSCLC in the model systems. Principal Component Analysis of RNAseq data from NSCLC tumor cell lines grown in the TMES, xenografts, and static 2D cultures and NSCLC PDX tumor cells grown in the TMES and PDXs. (**A**) PCA color coded by cell, (**B**) PCA color coded by growth condition. Heatmap of the Cancer Hallmark MSigDB gene set for (**C**) A549, (**D**) H1650, and (**E**) H1975 cells grown in the TMES, xenografts, and static 2D cultures. The heatmap color scales represents row-wise Z-scores.

We developed a NSCLC TMES using primary human microvascular ECs plated above a polycarbonate membrane with lung cancer associated fibroblasts and NSCLC cells co-plated below the membrane. Physiological hemodynamics derived from tumor terminal arterioles were applied to the ECs through a cone-and-plate viscometer while medium was continuously and independently perfused on the upper and lower sides of the membrane. For our studies, we used three NSCLC cell lines, A549 (RAS mutant G12S), H1650 (EGFR mutant exon 19 del(E746-A750)), and H1975 (EGFR mutant L858R and T790M), and two patient derived xenografts (PDXs), TM00199 (EGFR mutant L858R), and TM00219 (EGFR mutant del(E746-A750)) and T790M) that represent major genetic drivers of NSCLC^45^. We used next-generation sequencing of RNA (RNAseq) to compare the transcriptome of NSCLC cells grown under three different conditions, (1) 2D static cultures, (2) xenografts in athymic mice, and (3) the TMES (Fig. 1A), to determine in an unbiased manner if the NSCLC cells grown in the TMES are more like NSCLC cells grown in 2D static culture or the in vivo mouse xenografts. To enrich for tumor cells from the TMES, we used a negative plating mask that enables plating of cancer associated fibroblasts on 64% of the membrane while occluding fibroblasts from 9 precise 15 mm diameter areas that represent 36% of the membrane; these areas can then be cut at the end of an experiment allowing for rapid isolation and processing of an enriched population (> 95%) of tumor cells^25^.

We first explored the underlying structure of the RNAseq data using principal component analysis (PCA) to determine the relationships between the different tumor growth conditions (Fig. 1B,C). The PCA revealed that the tumor source is a primary factor in transcriptomic variability, and the growth environment is a secondary factor (Fig. 1B). When examining the different growth conditions by PCA, we find that for PC1 and PC2, the NSCLC cell lines grown in the TMES are closer to the xenografts than 2D static cultures (Fig. 1C), indicating that the TMES transcriptome is more in vivo*-*like than 2D static cultures. This is most apparent for A549 cells in PC1. For H1650 and H1975, the TMES is closer to the xenograft than 2D static cultures but the difference is more subtle than for A549. We attempted to culture cells from the PDXs in 2D and found that the cells rapidly died in culture. Thus, we were unable to adapt the PDX cells to 2D static conditions to perform a similar comparison of PDX cells grown in the three experimental conditions. Therefore, all work with the PDXs TM00199 and TM00219 are limited to the TMES and PDXs.

**Figure 2:**
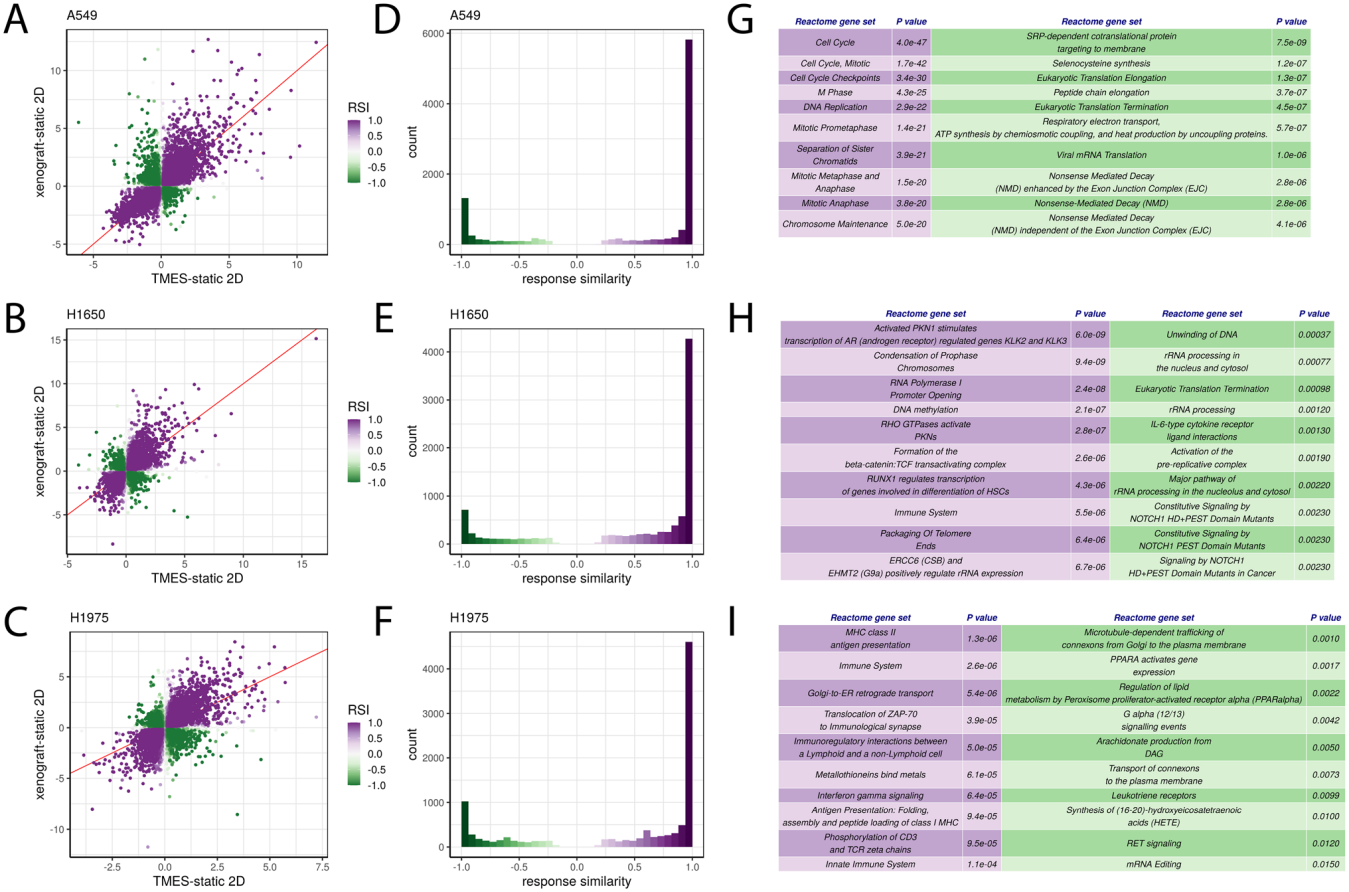
Response Similarity Analysis of NSCLC cell lines from the TMES and xenografts relative to 2D static cultures. (**A**–**C**) Xenograft vs TMES scatterplots log2 fold changes relative to static cell culture. Points are colored by their response similarity (RSI), where darker, more saturated colors indicate transcripts that show greater evidence for joint differential expression on both axes. Purple indicates transcripts that are concordantly regulated between the two conditions, whereas green indicates discordance. (**D**–**F**) Histograms of RSI values in (**A**–**C**). n = 6 for TMES, 2D static samples and xenografts. (**G**–**I**) Top ten concordantly (purple) and discordantly (green) regulated Reactome pathways.

The PCA was followed by gene set variability analysis. We focused on the Hallmark MSigDB gene set since this set is representative of gene signatures of broad and fundamental biological processes, many of which are often dis-regulated in cancer^46^. When examining the gene signature for oncogenesis using a clustered heatmap of the Hallmark MSigDB gene set for the NSCLC cell lines grown in the TMES, as xenografts, and in 2D static cultures, A549 (Fig. 1D) and H1975 (Fig. 1F) tumor cells from the TMES cluster with the xenografts. For H1650 (Fig. 1E), tumor cells from the TMES cluster with a subset of the 2D static cultures, as do the xenografts, indicating that for H1650 the Hallmarks gene set indicates that the TMES and 2D static cultures are similarly in-*vivo* like. Next, to explicitly identify aspects of shared differential biology in the transcriptome seen across the multiple experimental growth conditions (2D, TMES, xenograft), we employed a statistical intersection–union test. We identified both concordant (same direction) and discordant (opposite direction) instances of differential expression exhibited between the TMES to 2D contrast and xenograft to 2D contrast (Fig. 2). The asymmetry of the distribution of response similarity index (RSI) values across the transcriptome demonstrates that the PDX and the TMES models showed strong concordance and relatively little discordance relative to the 2D static model. Furthermore, a pathway analysis based on the Reactome database, which provides a much finer pathway-level perspective than the Hallmarks gene set, shows that the concordantly regulated genes have a higher degree of functional coherence than the discordantly regulated genes (Fig. 2G,H,I). This pattern was observed again using a second pathway database, KEGG (Supplemental Fig. 1). This demonstrates that the TMES across all three NSCLC lines recapitulates many of the transcriptional characteristics of a xenograft model that distinguishes it from 2D static cell culture.

**Figure 3:**
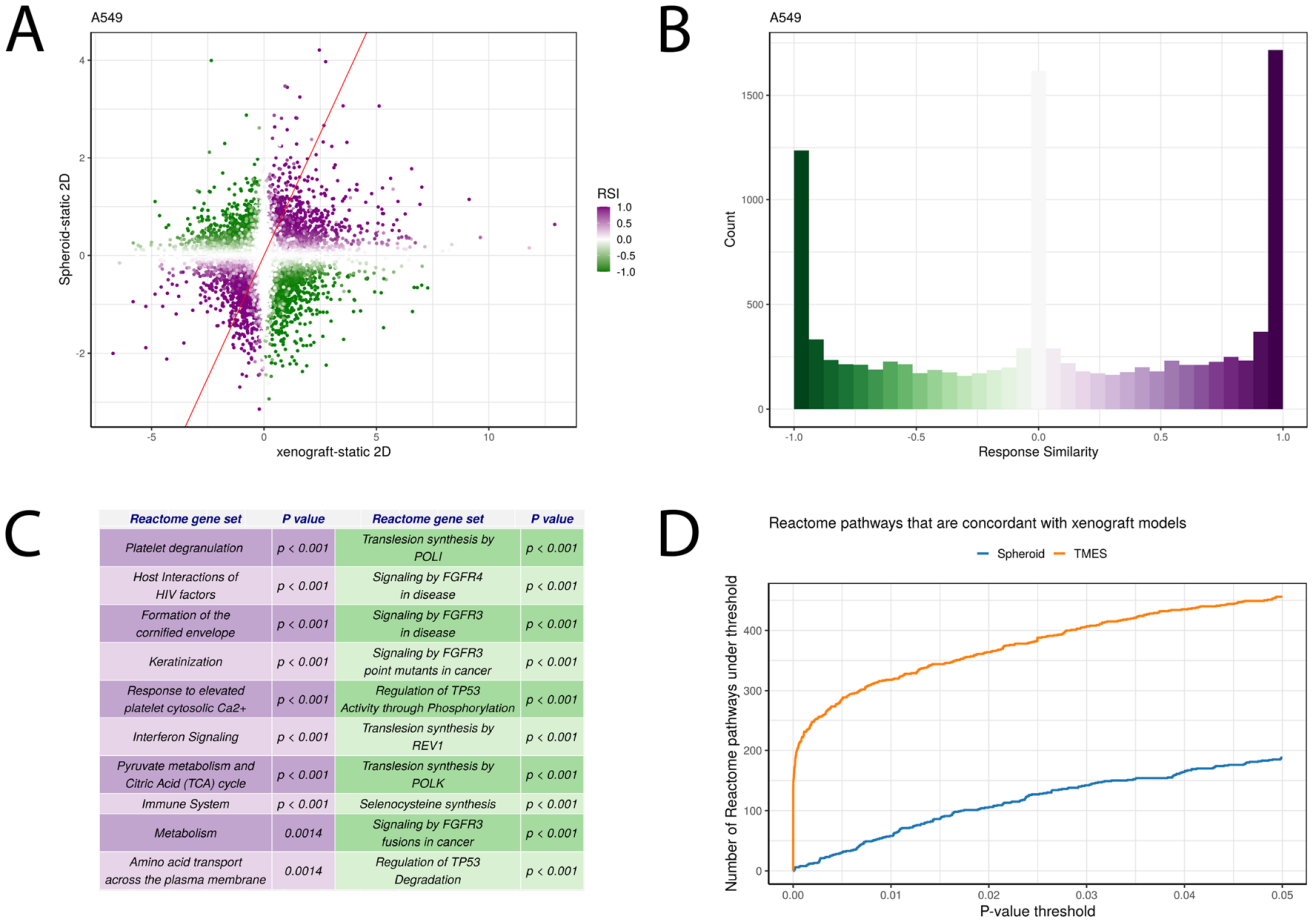
Transcriptome analysis of A549 spheroids relative to xenografts, 2D static cultures, and the TMES. (**A**) Spheroid vs xenografts scatterplots log2 fold changes relative to static cell culture. Points are colored by their response similarity (RSI), where darker, more saturated colors indicate transcripts that show greater evidence for joint differential expression on both axes. Purple indicates transcripts that are concordantly regulated between the two conditions, whereas green indicates discordance. (**B**) Histograms of RSI values in (**A**). (**C**) Plot of the number of Reactome pathways concordant with xenografts at p values up to 0.05.

Previous studies have shown that changing cell culture systems from 2 to 3D has profound effects on the transcriptomic state^38,47–50^. To explore the impact of transitioning to 3D on reproducing the xenograft transcriptomic state, we performed a statistical intersection–union test on A549 in 2D static cultures and 3D spheroids generated by the hanging droplet method^38^. We identified concordant and discordant occurrences of differential expression between the 3D spheroid to 2D contrast and xenograft to 2D contrast (Fig. 3A,B). We observed > 1500 concordantly regulated genes (RSI near 1), suggesting that large portions of the transcriptomic changes induced by xenograft conditions are recapitulated by 3D spheroids; however, 3D spheroids also produced nearly as many changes that were discordant with the xenograft state. The corresponding analysis comparing the TMES to xenografts shows that the transcriptomic changes induced by the TMES are more xenograft-like than those induced by 3D spheroid culture, both in terms of number and proportion of concordantly regulated genes (Fig. 2A,D,G). The TMES also appears more xenograft-like at the pathway-level. An analysis comparing the overrepresentation of high RSI values in Reactome pathways shows that the pathway-level changes induced by the xenograft state are more similar to the TMES than 3D spheroids (Fig. 3C). In this analysis, the TMES state has hundreds more concordant pathways than 3D spheroids at a wide range of significance thresholds. By using the changes induced by the transition from 2D static culture to xenografts as a benchmark, these results show that the TMES broadly induces a more in vivo-like transcriptional program than 3D spheroids.

**Figure 4:**
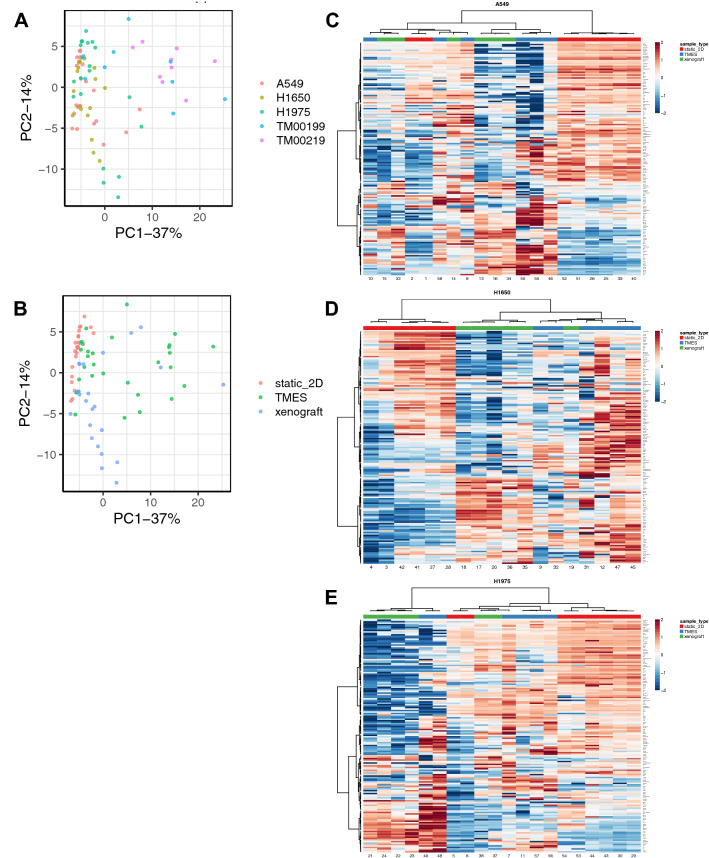
Protein Signal Pathway Analysis of NSCLC in the model systems. Principal Component Analysis of RPPA data from NSCLC tumor cell lines grown in the TMES, xenografts, and static 2D cultures and NSCLC PDX tumor cells grown in the TMES and PDXs. (**A**) PCA color coded by cell, (**B**) PCA color coded by growth condition. Heatmap and cluster analysis of RPPA from (**C**) A549, (**D**) H1650, (**E**) 1975 grown in the TMES, xenografts, and static 2D cultures. The heatmap color scales represents row-wise Z-scores.

To further delineate whether the NSCLC cells grown in the TMES are more like NSCLC cells grown in 2D static culture or in vivo mouse xenografts, we used reverse phase protein array (RPPA) to compare the proteome and phosphoprotein-based signaling architecture of NSCLC cell lines grown in the TMES to in vivo NSCLC xenografts and in vitro 2D static cultures (Fig. 4). At the functional signaling level the individual tumor was not the major source of variability (Fig. 4A). Rather, the experimental condition was a driver of the overall protein signaling landscape (Fig. 4B). In PC1 the NSCLCs 2D static cultures cluster on the left whereas the TMES and xenografts are scattered towards the right, occupying similar space. We next applied hierarchical clustering to the RPPA signaling data from the individual NSCLC cell lines in the three experimental systems (2D, TMES, xenograft). For H1650 (Fig. 4D), the tumors from the TMES cluster with the xenografts. For A549 (Fig. 4C) and H1975 (Fig. 4E), tumor cells from the TMES also cluster with the xenografts; however, for both A549 and H1975 there is a subset of 2D static cultures that cluster with the TMES and xenografts. This data suggests that overall there is concordance between the TMES and xenograft at the functional signaling level, further indicating that tumor cells in the TMES are in vivo*-*like. When examining the top differentially expressed proteins/phosphoproteins from the RPPA across the three NSCLC cell lines (Supplemental Figs. 2, 3, and 4) in the three different model systems, TMES, xenograft, and 2D static culture, there is an increase in BAD pS112 and a decrease in cleaved caspase 3. This suggests that there is less apoptosis in tumor cells in the TMES and xenograft compared to 2D static cultures.

**Figure 5:**
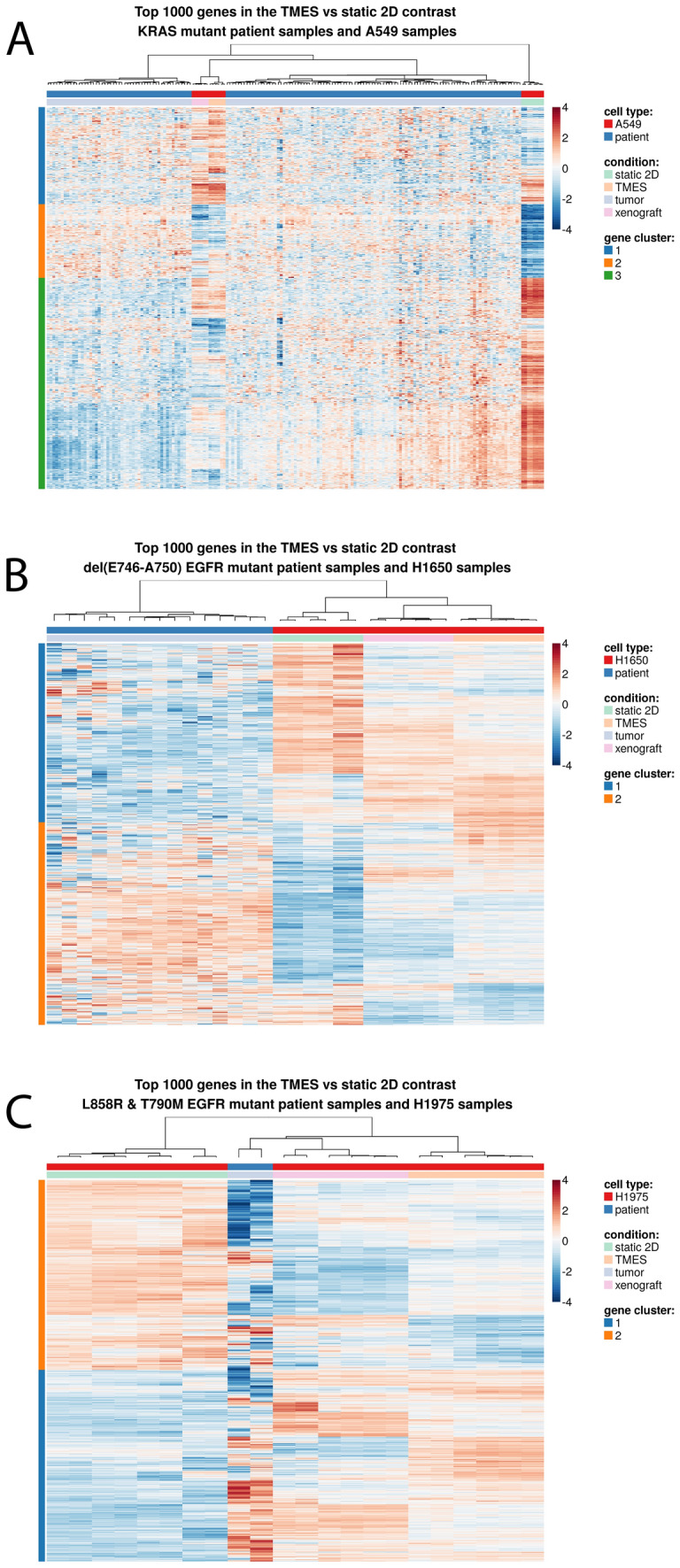
Patient transcriptome similarity to the TMES. Heatmap analysis of the top 1000 genes from the TMES vs 2D static culture contrast of patients in the NSCLC TCGA with the corresponding driver mutation and the three different model systems; TMES, xenograft, and 2D static culture. (**A**) A549, (**B**) H1650, and (**C**) 1975. The heatmap color scales represents row-wise Z-scores.

The above data all indicate that NSCLC cell lines grown in the TMES are more similar to xenografts than 2D static culture; however, a critical test is how similar the gene expression profiles of the NSCLC cells in the TMES are to patient tumors. We contrasted the gene expression of patients in the NSCLC TCGA with the corresponding driver mutation (RAS mutant for A549, EGFR mutant exon 19 del(E746-A750) for H1650, and EGFR mutant L858R and T790M for H1975) to NSCLC cells grown in the three different model systems, TMES, xenograft, and 2D static culture, using the top 1000 differentially expressed genes between the TMES and static 2D cultures. The A549 TMES and xenograft models cluster together with KRAS mutant NSCLC patient tumors (Fig. 5A), while the 2D static cultures clusters away, indicating that the A549 transcriptome is similar to patients when grown in the TMES and as a xenograft. For H1650 (Fig. 5B), the transcriptome of patients with the del(E746-A750) EGFR mutation cluster separately from H1650 cells in the three different model systems. There were only two patients in the NSCLC TCGA with both the L858R and T790M EGFR mutation; H1975 (Fig. 5C) TMES and xenografts cluster with those two patients and away from the 2D static cultures. The patient heterogeneity of NSCLC tumors notwithstanding, two of the three NSCLC cell lines grown in the TMES cluster with patient samples over 2D static cultures.

**Figure 6:**
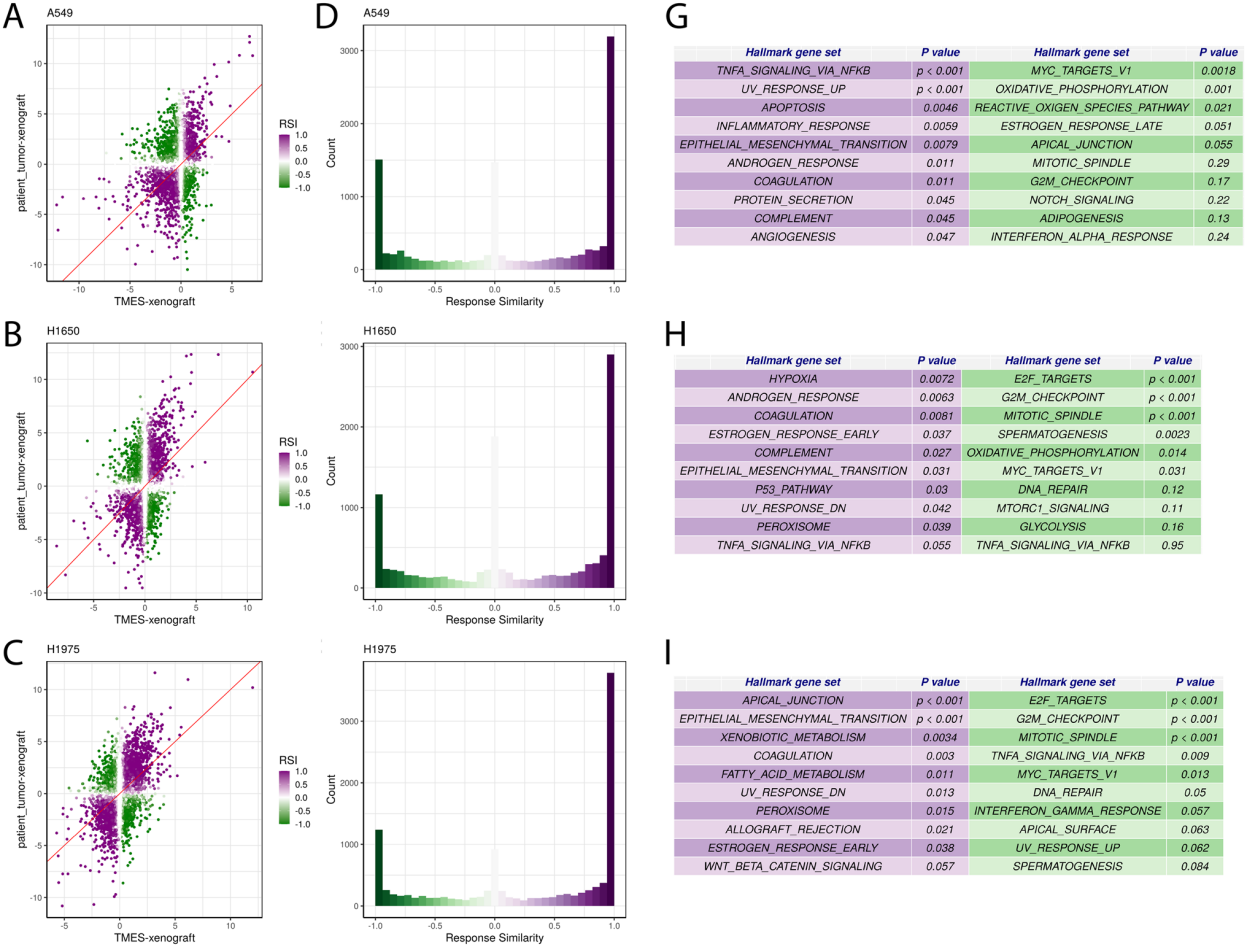
Response Similarity Analysis of NSCLC cell lines relative to patient tumors. (**A**–**C**) Xenograft vs TMES scatterplots log2 fold changes relative to patient tumors with the same genetic driver mutation. Points are colored by their response similarity (RSI), where darker, more saturated colors indicate transcripts that show greater evidence for joint differential expression on both axes. Purple indicates transcripts that are concordantly regulated between the two conditions, whereas green indicates discordance. (**D**–**F**) Histograms of RSI values in (**A**–**C**). (**G**–**I**) Top ten concordantly (purple) and discordantly (green) regulated hallmark pathways.

To explore the comparison of tumor cells in the TMES with patient tumors further, we explicitly asked if tumor cells in the TMES or in xenografts are more similar to the patient transcriptome. We again employed a statistical intersection–union test to identify both concordant and discordant differential expression. We compared the gene expression of patients in the NSCLC TCGA with the corresponding driver mutation (RAS mutant for A549, EGFR mutant exon 19 del(E746-A750) for H1650, and EGFR mutant L858R for H1975) to NSCLC cells grown in the TMES or as a xenograft (Fig. 6). We found that in terms of deviations from xenograft samples, patient tumors and TMES samples are more similar than different (Fig. 6D–F), with many transcripts showing highly concordant responses (Fig. 6A–C). A pathway analysis based on the Hallmark gene sets used above shows that concordantly and discordantly regulated genes have limited functional coherence (Fig. 6G–I). These data further demonstrate that the TMES recapitulates many of the transcriptional characteristics of patient tumors, and suggests that the TMES may better reflect the patient biological state than xenografts.

**Figure 7:**
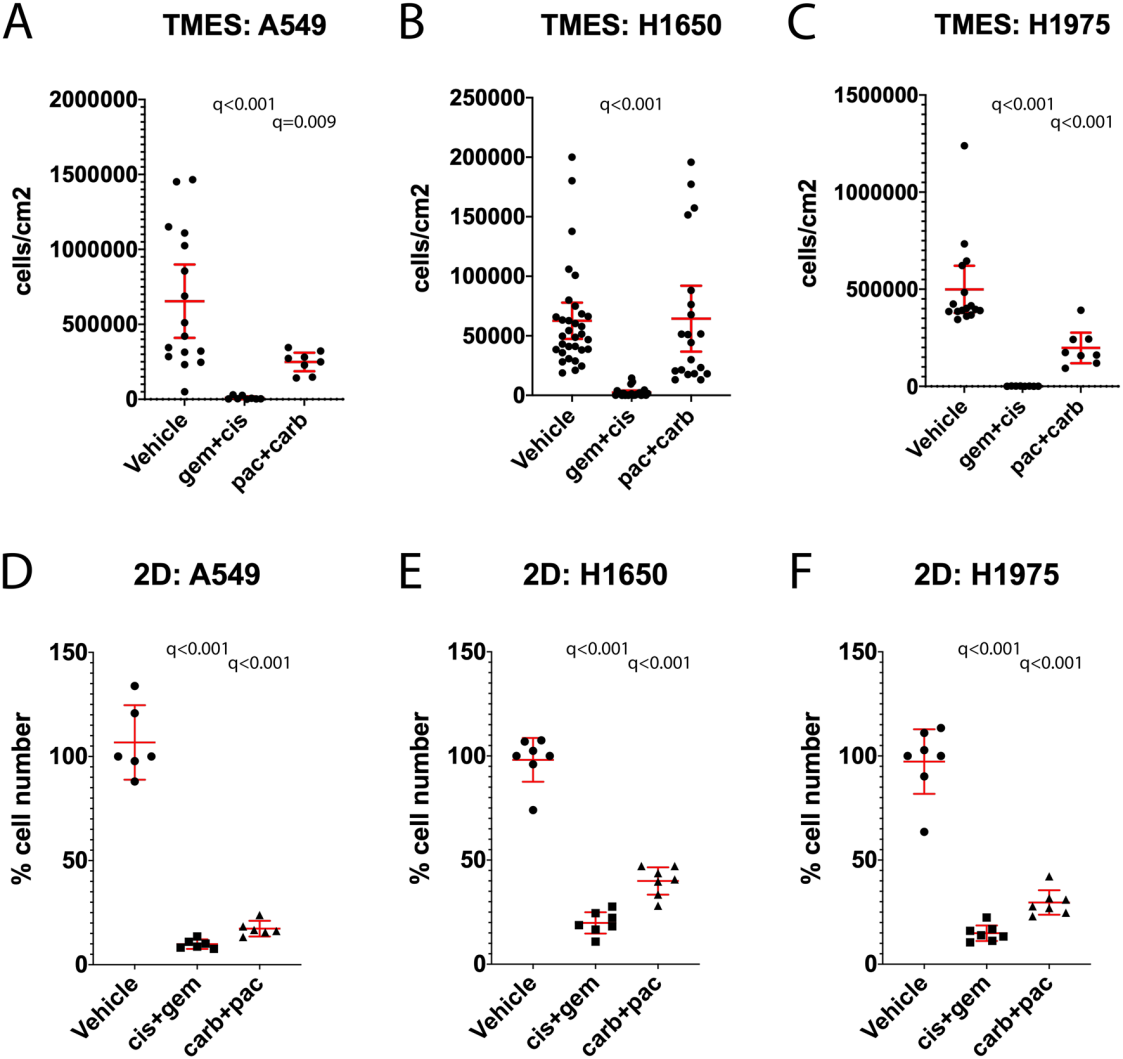
The NSCLC TMES responds to cytotoxic chemotherapies at human patient doses. NSCLC cells from A549 (**A**), H1650 (**B**,**D**), and H1975 (**C**,**E**) were grown in the TMES for four days and then dosed with either 35.4 μM gemcitabine and 6.5 μM cisplatin, 6.2 μM paclitaxel and 67 μM carboplatin for 3 days. Tumor cell number was determined using luciferase signal as a surrogate for cell number with a standard curve. Plotted are individual measurements of tumor cells across multiple TMES device runs with the mean and 95% confidence intervals shown in red. ANOVA followed by two stage step up method of Benjamini, Krieger and Yekutieli to control for the FDR, relative to control; q values reported on the graphs.

The transcriptomic analysis collectively indicates that tumor cells grown in the TMES reflect the biology of NSCLC patient tumors. This leads to the prediction that tumor cell growth in the TMES should be controlled using standard of care chemotherapeutics at concentrations equivalent to typical concentration attained in patients. We evaluated the efficacy of two standard of care chemotherapeutic combinations used for the treatment of NSCLC; the combination of gemcitabine (35.4 μM) and cisplatin (6.5 μM), and the combination of paclitaxel (6.2 μM) and carboplatin (67 μM) on the three NSCLC cell lines in the TMES (Fig. 7). The drug concentrations used in our studies were based on the patient dosing in the INTACT1 and INTACT2 clinical trials^51,52^. Patients in the INTACT1 trial were treated with 1250 mg/m(2), IV/30 min infusion of gemcitabine and 80 mg/m(2) of cisplatin yielding a plasma C_max_ of 35.4 μM gemcitabine and a C_max_ of 6.5 μM cisplatin^51,53–56^. For paclitaxel and carboplatin, patients in the INTACT2 trial were dosed with 225 mg/m(2), IV/3 hr infusion of paclitaxel and carboplatin was dosed at area under concentration/time curve of 6 mg/min/mL^52^. This generated a plasma C_max_ of 6.2 μM for paclitaxel and 67 μM for carboplatin^54,57–59^. As we have done previously, we assessed the efficacy of these drug combinations on growth, following three days of drug exposure after tumor cell biology had been established in the TMES^25^. The drug combinations were added to the inflow media on the endothelial cell side under conditions of tumor hemodynamic flow and transport. Gemcitabine and cisplatin significantly inhibited growth as measured by cell number in A549, H1650, and H1975 in the TMES (Fig. 7A,B,C). The growth inhibition of paclitaxel and carboplatin is more variable across the cell lines, significantly inhibiting growth in A549 and H1975 in the TMES (Fig. 7A,C). The combination of gemcitabine plus cisplatin was more effective than paclitaxel plus carboplatin in the TMES in all three NSCLC lines. The cytotoxic drug combinations of gemcitabine plus cisplatin and paclitaxel plus carboplatin were all effective in 2D static cultures displaying a less heterogeneous response than in the TMES (Fig. 7D,E,F).

**Figure 8:**
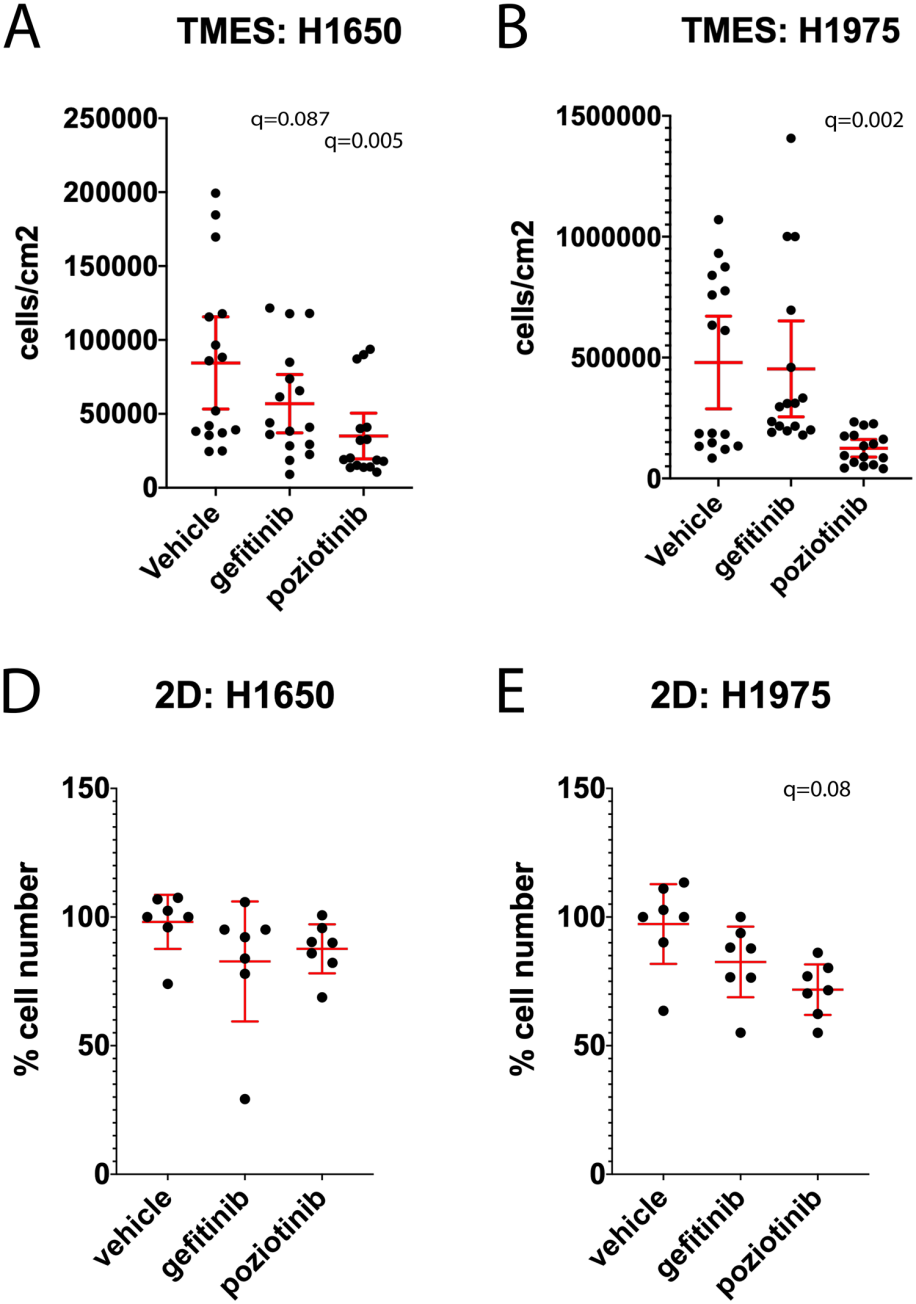
The NSCLC TMES responds to EGFR inhibitors according to EGFR mutational status. NSCLC cells from H1650 (**A**, **C**) and H1975 (**B**, **D**) were grown in the TMES for four days and then dosed with either 848 nM gefitinib or 172 nM poziotinib. Tumor cell number was determined using luciferase signal as a surrogate for cell number with a standard curve. Plotted are individual measurements of tumor cells across multiple TMES device runs with the mean and 95% confidence intervals shown in red. ANOVA followed by two stage step up method of Benjamini, Krieger and Yekutieli to control for the FDR, relative to control; q values reported on the graphs.

We evaluated the effectiveness of one FDA approved EGFR inhibitor, gefitinib (848 nM), and one investigational EGFR inhibitor, poziotinib (172 nM), on the two NSCLC cell lines that carry EGFR mutations: H1650 (EGFR mutant exon 19 del(E746-A750)), and H1975 (EGFR mutant L858R and T790M) (Fig. 8). In the INTACT1 and INTACT2 clinical trials, gefitinib was dosed orally once per day at 250 mg/day. This generates a plasma Cmax of 848 nM gefiitinb^51,52,60,61^. Pharmacokinetic analysis of poziotinib/HM781-36B, the 3^rd^ generation EGFR inhibitor that is active against the T790M mutant EGFR, demonstrated that a 16 mg/day oral dose generates a plasma Cmax of 172 nM poziotinib^62^. As is predicated by the genotype, gefitinib and poziotinib significantly inhibited growth of H1650 in the TMES, whereas only poziotinib significantly inhibited the growth of H1975 in the TMES (Fig. 8A,B). This indicates that the effectiveness of EGFR inhibitors on NSCLC cells in the TMES corresponds with EGFR mutational status. This was specific to the TMES; parallel 2D static experiments using gefitinib and poziotinib did not inhibit H1650 growth (Fig. 8C,D), whereas poziotinib did inhibit H1975 2D static growth; however the differential effect of gefitinib and poziotinib on 2d static growth in H1975 is difficult to interpret when gefitinib failed to inhibit H1650 2S static growth.

## Discussion

Here we show that the NSCLC TMES reproduces the in vivo transcriptional and proteomic program and responds to chemotherapeutics and molecularly targeted therapies at concentrations that correspond to therapeutic plasma levels in humans. Importantly, NSCLC cells in the TMES, in contrast to 2D static cultures, respond to EGFR inhibitors according to the EGFR mutational status of the NSCLC cells.

Mutations in the tyrosine kinase domain of the EGFR correlate with sensitivity to EGFR tyrosine kinase inhibitors; the presence of activating mutations in the EGFR, such as the E746-A750 in-frame deletion and the L858R substitution, are associated with clinical sensitivity to EGFR inhibitors^63–65^. The EGFR inhibitor gefitinib has superior response rates and progression free survival when compared to conventional chemotherapeutics in patients with activating mutations in EGFR^66^. Unfortunately, these clinical responses are not durable. Resistance is typically mediated by reactivation of EGFR signaling with over half of the cases of acquired resistance being mediated by the T790M mutation in the EGFR, which reduces the binding affinity of the first-generation EGFR inhibitors such as gefitinib^67^. We found that in the TMES, using concentrations that correspond to human therapeutic plasma levels, gefitinib was effective against the NSCLC cell line H1650 that carries the EGFR mutant exon 19 del(E746-A750), and that the NSCLC line carrying the T790M EGFR mutation, H1975, was insensitive to gefitinib. We further observed that poziotinib was effective against the T790M EGFR mutation NSCLC line H1975 and that poziotinib was more effective than gefitinib in the TMES, consistent with previous pre-clinical model experiments^68^. Poziotinib had low clinical activity in acquired resistant NSCLC, including in patients that carried T790M mutations^69^, and poziotinib recently failed to meet a primary endpoint in a clinical trial focused on EGFR exon 20 mutation^70,71^. The clinical failures of poziotinib, and other irreversible EGFR inhibitors^72–74^, on patients previously treated with EGFR inhibitors may be a result of dose limiting toxicities which are difficult to readout in preclinical cancer models. The more relevant setting for poziotinib may be in untreated patients with activating EGFR mutations. Subsequent acquired resistant mutations, such as the T790M with elevated affinity for ATP^75^, may be better treated with T790M mutant-selective EGFR inhibitors.

In the TMES we observed that both chemotherapeutic combinations, gemcitabine plus cisplatin and paclitaxel plus carboplatin, were effective when used at concentrations that correspond to human therapeutic plasma levels. The combination of gemcitabine plus cisplatin was more effective than paclitaxel plus carboplatin, consistent with the combination of gemcitabine and cisplatin being indicated in patients with better performance status^76,77^. The response of NSCLC in the TMES to therapies at therapeutic plasma levels indicates that the TMES may have applicability to evaluate the effectiveness of cancer therapies. Testing additional NSCLC cells and drugs in the system will ultimately determine the effectiveness of the TMES for predicting NSCLC therapeutic efficacy.

We found that a single ‘-omic’ measure is incomplete and that determining the molecular state on multiple levels is necessary to determine the molecular features that drive any given biological state. When comparing the transcriptome and proteome of the NSCLC cells in the TMES to the in vivo xenograft and NSCLC TCGA patient data we found that the variance in transcriptional measurements between tumor types was much greater than the variance between experimental growth conditions, whereas the variance in proteomic data was more balanced across tumors types and growth conditions. Using the Hallmarks gene set, the xenograft and the TMES models cluster together for A549 and H1975 NSCLC cells indicating that the transcriptome of cancer driver pathways is similar in these two NSCLC lines when grown in the TMES and as a xenograft. For H1650, the transcriptome defined by the Hallmarks gene set does not distinguish the different model systems. However, the functional signaling and proteome defined by RPPA for xenografts and TMES models cluster together for H1650. For A549 and H1975, the proteome in tumor cells from xenograft and the TMES predominately cluster together, but heterogeneity in the cell signaling architecture generates imperfect separation of samples by growth condition. Interestingly, when we directly tested if NSCLC tumor cells grown in the TMES were more like in vivo xenografts than 2D static cultures using a statistical intersection–union test, we found many more concordant biologies with functional coherence between the TMES and xenograft than 2D static and xenograft indicating that the TMES is more in vivo-like than 2D static cultures. This observation extends to 3D spheroids, with the TMES more in vivo-like than 3D spheroids. While it is important to note that a portion of the TMES in vivo-like biology is driven by the 3D architecture, the inclusion of ECs experiencing physiologic shear, cancer associated fibroblasts (CAFs), and flow pushes tumor cells into an even more in vivo-like state. Previously we showed that loss of mechanical shear and flow led to death of the ECs and loss of physiologic drug response^25^.

These general observations hold true when comparing the NSCLC TMES transcriptome to the patient NSCLC TCGA transcriptome. The xenograft and the TMES models cluster together with patient tumors for A549 and H1975 NSCLC cells but for H1650, the transcriptome of patients with the del(E746-A750) EGFR mutation cluster separately from the three different model systems. This is consistent with the H1650 functional signaling and proteome being more of a measure of in vivo*-*like biology than the transcriptome. This also raises the issue that certain tumor subtypes with specific driver mutations may be more effectively modeled in the TMES than others. When we explicitly tested if the transcriptome of tumor cells in the NSCLC TCGA with the corresponding driver mutation to tumor cells grown in the TMES or a xenograft was more alike using a statistical intersection–union test, we found that tumor cells in the TMES were more similar to patient tumors than the xenografts. This is striking given the heterogeneity of NSCLC patient tumors. A pathway analysis of concordant and discordant transcripts did not provide any significant insight into the underlying biology driving TMES tumor cells similarity to patient tumors, likely a result of patient tumor heterogeneity where there are limited gene sets of functional coherence across large patient populations. This parallels our observation from the PCA analysis indicating that the donor is the major driver of the underlying transcriptomic state.

There are a multitude of 3D in vitro models of the tumor microenvironment, including spheroids and organoids, engineered scaffolds, microfluidics, and ex vivo tumor slices or chips^78,79^. Each unique model system possesses inherent advantages and disadvantages for addressing a given question. Our prior work and the work presented herein suggests that the TMES recreates multiple aspects of cancer biology. These include tumor growth, the in vivo xenograft and patient transcriptional program, response to chemotherapeutics at concentrations that correspond to human therapeutic plasma levels, and responses to chemotherapeutics that parallel patient’s responses. This is consistent with the substantial success of parallel platforms to model vascular health and disease, and rare metabolic disorders^37,80–84^. The TMES is currently based on a 75 mm transwell, which while providing substantial material and allowing for high content analytics, it does not provide high throughput. Along the drug discovery pipeline, the TMES may best function in de-risking and development of lead compounds as opposed to screening, which may be better served by 3D spheroid technologies. The inclusion in the TMES of ECs exposed to physiologic mechanical forces and the continual perfusion of media offer advantages that can be leveraged, including dosing drugs from the EC side as we have done in this study. Our TMES incorporates a 3D scaffold, physiological matrices, multiple cell types, patient-derived cells, and perfusion in an orientation that recreates many aspects of the in vivo tumor architecture, biological transport, and tumor-specific hemodynamics^25^. By demonstrating that the TMES can be applied to another solid tumor type, NSCLC, we show the broader applicability of the system that suggests the TMES can be used to advance the discovery and development of effective anticancer agents for a variety of tumor types.

## Supplementary Information

Supplementary Information 1.

Supplementary Information 2.
