# Supplementary material for: Validation of a multicellular tumor microenvironment system for modeling patient tumor biology and drug response
Source: Sci Rep. 2021 Mar 10;11:5535. doi: 10.1038/s41598-021-84612-z (PMC7946945; doi:10.1038/s41598-021-84612-z)
Supplement: Supplementary file 1 — Supplementary Information 1. [file 41598_2021_84612_MOESM1_ESM.pdf]

A

KEGG gene set	Q value	KEGG gene set	Q value
DNA replication	6.8e-14	Oxidative phosphorylation	2.3e-06
Complement and coagulation cascades	5.2e-09	Ribosome	2.3e-06
Mismatch repair	4.1e-07	N-Glycan biosynthesis	5.1e-04
Cell cycle	4.1e-07	Alzheimer's disease	8.8e-04
Nucleotide excision repair	1.3e-06	Parkinson's disease	6.3e-03
Pyrimidine metabolism	5.0e-06	Phosphatidylinositol signaling system	1.4e-02
Spliceosome	1.4e-05	Fc gamma R-mediated phagocytosis	2.9e-02
Homologous recombination	1.8e-05	Steroid biosynthesis	7.2e-02
RNA transport	4.5e-05	Huntington's disease	8.1e-02
Drug metabolism - cytochrome P450	1.7e-04	Epithelial cell signaling in Helicobacter pylori infection	8.1e-02

B

KEGG gene set	Q value	KEGG gene set	Q value
Leishmaniasis	1.0e-05	Chemokine signaling pathway	1
Graft-versus-host disease	1.0e-05	Folate biosynthesis	1
Type I diabetes mellitus	1.2e-05	Regulation of autophagy	1
Allograft rejection	1.6e-05	Glyoxylate and dicarboxylate metabolism	1
Autoimmune thyroid disease	3.1e-05	Glycolysis / Gluconeogenesis	1
Cell adhesion molecules (CAMs)	8.6e-05	Renin-angiotensin system	1
Systemic lupus erythematosus	2.7e-04	Propanoate metabolism	1
Staphylococcus aureus infection	4.1e-04	Bacterial invasion of epithelial cells	1
Antigen processing and presentation	4.1e-04	Ribosome	1
Rheumatoid arthritis	1.4e-03	Nitrogen metabolism	1

C

KEGG gene set	Q value	KEGG gene set	Q value
Systemic lupus erythematosus	1.8e-10	Ribosome	0.41
Allograft rejection	3.7e-08	Circadian rhythm	0.70
Autoimmune thyroid disease	5.9e-08	Epithelial cell signaling in Helicobacter pylori infection	0.70
Staphylococcus aureus infection	3.2e-07	Phosphatidylinositol signaling system	0.70
Graft-versus-host disease	1.0e-06	Regulation of autophagy	0.70
Type I diabetes mellitus	1.0e-06	Inositol phosphate metabolism	0.70
Antigen processing and presentation	3.7e-06	Ribosome biogenesis in eukaryotes	0.70
Asthma	6.0e-05	Glycerophospholipid metabolism	1.00
Complement and coagulation cascades	3.0e-04	Biosynthesis of unsaturated fatty acids	1.00
Viral myocarditis	3.9e-04	Fatty acid elongation	1.00

Supplemental Figure 1
