# Supplementary material for: Validation of a multicellular tumor microenvironment system for modeling patient tumor biology and drug response
Source: Sci Rep. 2021 Mar 10;11:5535. doi: 10.1038/s41598-021-84612-z (PMC7946945; doi:10.1038/s41598-021-84612-z)
Supplement: Supplementary file 2 — Supplementary Information 2. [file 41598_2021_84612_MOESM2_ESM.pdf]

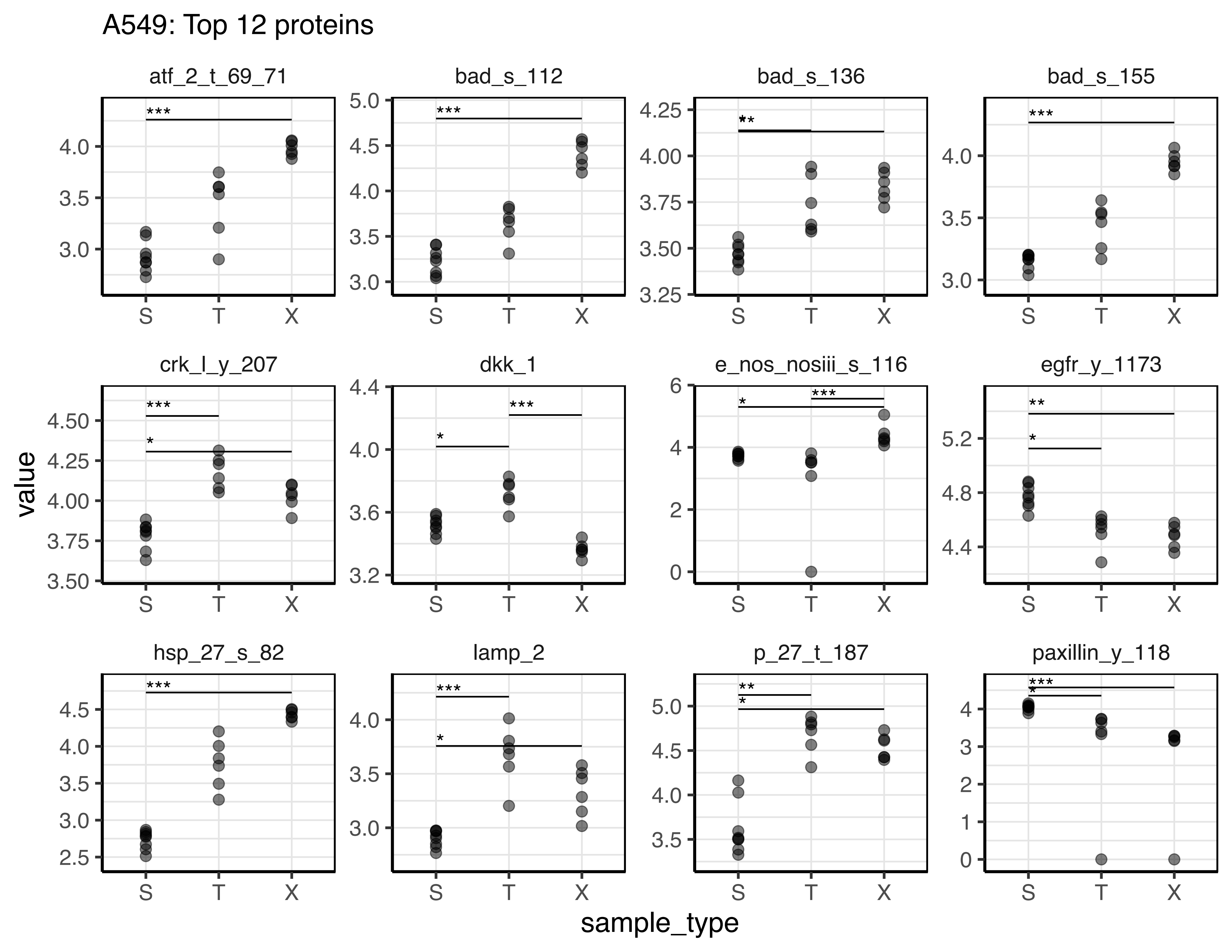
A549: Top 12 proteins
atf_2_t_69_71
bad_s_112
bad_s_136
bad_s_155
crk_l_y_207
dkk_1
e_nos_nosiii_s_116
egfr_y_1173
hsp_27_s_82
lamp_2
p_27_t_187
paxillin_y_118
value
sample_type
S
T
X

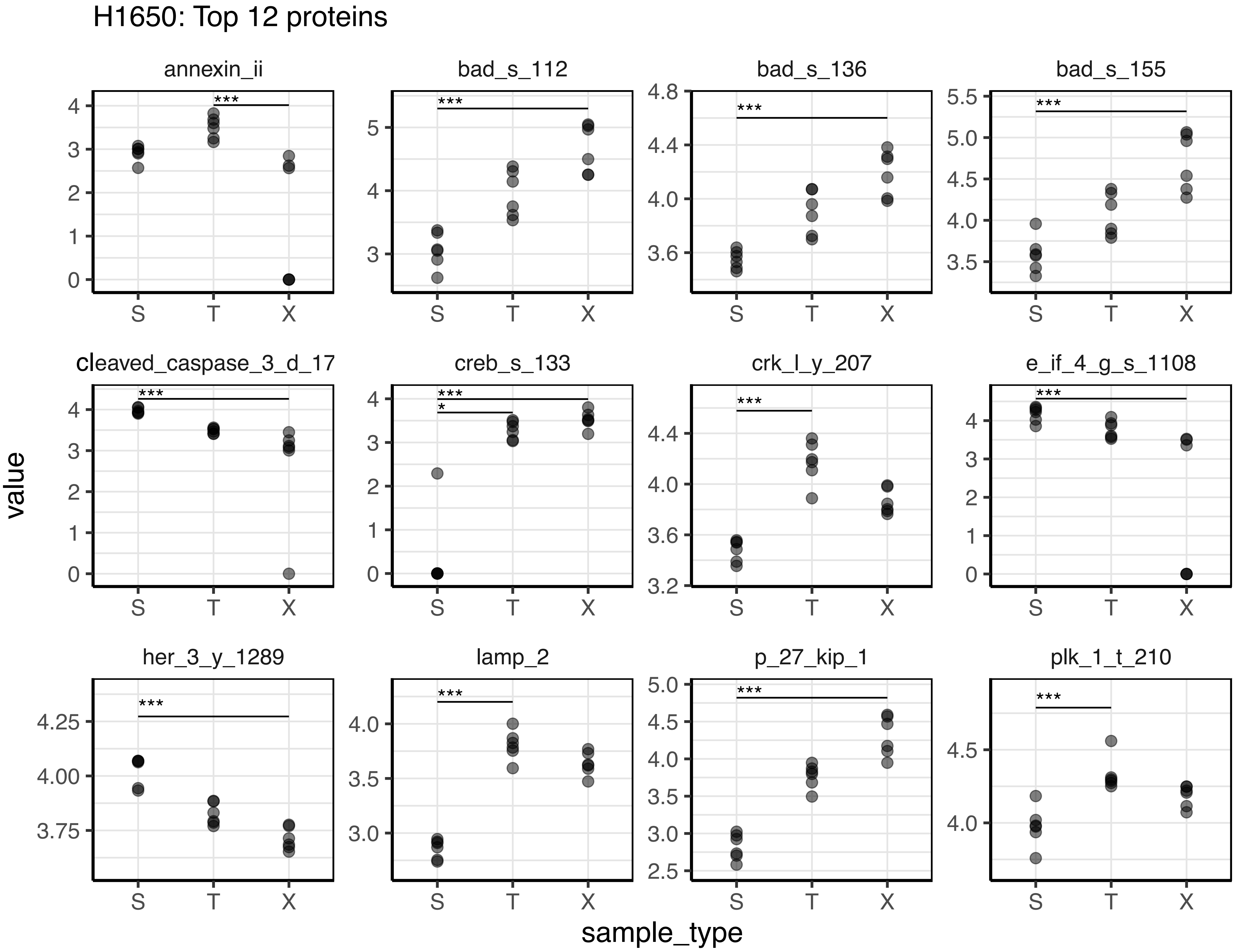
H1650: Top 12 proteins
annexin_ii
bad_s_112
bad_s_136
bad_s_155
cleaved_caspase_3_d_17
creb_s_133
crk_l_y_207
e_if_4_g_s_1108
her_3_y_1289
lamp_2
p_27_kip_1
plk_1_t_210
value
sample_type
S
T
X
***
*

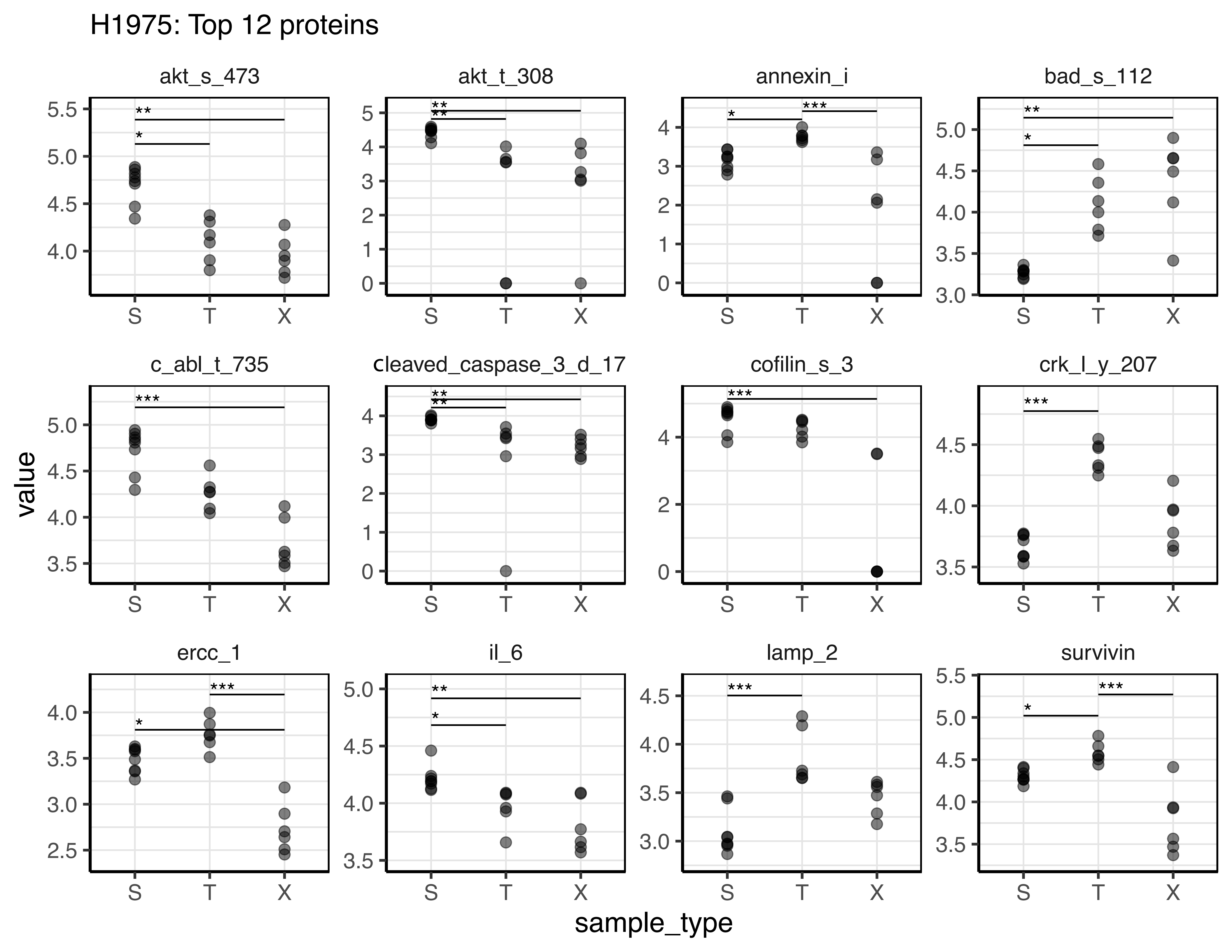
H1975: Top 12 proteins
akt_s_473
akt_t_308
annexin_i
bad_s_112
c_abl_t_735
cleaved_caspase_3_d_17
cofilin_s_3
crk_l_y_207
ercc_1
il_6
lamp_2
survivin
value
sample_type
S
T
X
